# Development of an invasively monitored porcine model of acetaminophen-induced acute liver failure

**DOI:** 10.1186/1471-230X-10-34

**Published:** 2010-03-30

**Authors:** Philip N Newsome, Neil C Henderson, Leonard J Nelson, Costas Dabos, Celine Filippi, Chris Bellamy, Forbes Howie, Richard E Clutton, Tim King, Alistair Lee, Peter C Hayes, John N Plevris

**Affiliations:** 1 Centre for Liver Research, Institute of Biomedical Research, University of Birmingham, Birmingham B15 2TT, UK; 2 Centre for Liver and Digestive Disorders, Royal Infirmary of Edinburgh, Edinburgh, EH16 4SB, UK; 3 Department of Pathology, Royal Infirmary of Edinburgh, Edinburgh, EH16 4SB, UK; 4 Department of Veterinary Clinical Studies, Royal (Dick) School of Veterinary Studies, The University of Edinburgh, Edinburgh, EH25 9RG, UK; 5 Roslin Institute, Roslin, UK; 6 Department of Anaesthetics, Royal Infirmary of Edinburgh, Edinburgh, EH16 4SB, UK

## Abstract

**Background:**

The development of effective therapies for acute liver failure (ALF) is limited by our knowledge of the pathophysiology of this condition, and the lack of suitable large animal models of acetaminophen toxicity. Our aim was to develop a reproducible invasively-monitored porcine model of acetaminophen-induced ALF.

**Method:**

35kg pigs were maintained under general anaesthesia and invasively monitored. Control pigs received a saline infusion, whereas ALF pigs received acetaminophen intravenously for 12 hours to maintain blood concentrations between 200-300 mg/l. Animals surviving 28 hours were euthanased.

**Results:**

Cytochrome p450 levels in phenobarbital pre-treated animals were significantly higher than non pre-treated animals (300 vs 100 pmol/mg protein). Control pigs (n = 4) survived 28-hour anaesthesia without incident. Of nine pigs that received acetaminophen, four survived 20 hours and two survived 28 hours. Injured animals developed hypotension (mean arterial pressure; 40.8 +/- 5.9 vs 59 +/- 2.0 mmHg), increased cardiac output (7.26 +/- 1.86 vs 3.30 +/- 0.40 l/min) and decreased systemic vascular resistance (8.48 +/- 2.75 vs 16.2 +/- 1.76 mPa/s/m3). Dyspnoea developed as liver injury progressed and the increased pulmonary vascular resistance (636 +/- 95 vs 301 +/- 26.9 mPa/s/m3) observed may reflect the development of respiratory distress syndrome.

Liver damage was confirmed by deterioration in pH (7.23 +/- 0.05 vs 7.45 +/- 0.02) and prothrombin time (36 +/- 2 vs 8.9 +/- 0.3 seconds) compared with controls. Factor V and VII levels were reduced to 9.3 and 15.5% of starting values in injured animals. A marked increase in serum AST (471.5 +/- 210 vs 42 +/- 8.14) coincided with a marked reduction in serum albumin (11.5 +/- 1.71 vs 25 +/- 1 g/dL) in injured animals. Animals displayed evidence of renal impairment; mean creatinine levels 280.2 +/- 36.5 vs 131.6 +/- 9.33 μmol/l. Liver histology revealed evidence of severe centrilobular necrosis with coagulative necrosis. Marked renal tubular necrosis was also seen. Methaemoglobin levels did not rise >5%. Intracranial hypertension was not seen (ICP monitoring), but there was biochemical evidence of encephalopathy by the reduction of Fischer's ratio from 5.6 +/- 1.1 to 0.45 +/- 0.06.

**Conclusion:**

We have developed a reproducible large animal model of acetaminophen-induced liver failure, which allows in-depth investigation of the pathophysiological basis of this condition. Furthermore, this represents an important large animal model for testing artificial liver support systems.

## Background

Acute liver failure (ALF) is a dramatic clinical syndrome that results from massive hepatic necrosis. The mortality of ALF remains high despite progress in the fields of intensive care management and liver transplantation [1]. ALF refers to the rapid development of severe acute liver injury with impaired synthetic function and encephalopathy in subjects with previously normal, or well-compensated liver disease [2]. Generation of a suitable animal model would allow for a greater understanding of the underlying pathophysiology of this condition and stimulate the development and refinement of treatments for ALF. There is a need for a suitable large animal model of hepatic injury to facilitate the development of bio-artificial liver support systems in humans and to assist pre-clinical assessment [3].

Since the recognition of acetaminophen-induced ALF in the 1960s, acetaminophen overdose has become the commonest cause of liver failure in the United Kingdom [4]. Although an effective antidote (*N*-acetylcysteine) has been available for many years, the late presentation of many cases ensures a significant number develop liver failure. Early large animal models of acetaminophen toxicity were unreliable, producing inconsistent toxicity from animal to animal. Gazzard et al [5] showed that dogs receiving oral doses greater than 1 g/kg died of nonhepatic causes, becoming cyanotic immediately before death with methylene blue-resistant methaemoglobinaemia. Human studies have shown that serum acetaminophen levels must be >140 mg/mL for greater than 20 hours for consistent hepatic necrosis to occur [6]. Using this, Francavilla et al [7] studied various doses and dosing schedules on 52 beagle dogs. These initially received 750 mg/kg acetaminophen subcutaneously followed by 200 mg/kg nine hours later, and then a further 200 mg/kg twenty-four hours later. Mortality rates in this group were: 0% at 24 hours, 10% at 48 hours, and 90% at 72 hours. Serum transaminase levels were greatly increased to 8206 +/- 3000 U/L at 48 hours and 21 253 +/- 3746 U/L at 72 hours. This correlated with liver biopsy samples that revealed severe centrilobular necrosis.

Several approaches have been adopted to control methaemoglobinaemia in animal models: Kelly et al [8] using dogs, suspected high plasma acetaminophen levels were responsible for the lethal methaemoglobinaemia that characterised their early experiments. They controlled acetaminophen infusion in an attempt to maintain its levels at 175 to 200 mg/mL for the first 20 hours. Methylene blue was administered at 10 mg/kg if blood samples appeared brown on visual inspection. Pilot data showed that levels of acetaminophen greater than 300 mg/mL were associated with severe methemoglobinemia.

Cellular glutathione (GSH) levels must be depleted to approximately 20% of their original levels before toxic acetaminophen damage can occur, [9,10]. To reduce variation in the acetaminophen dose required and the time taken for the development of signs, Kelly et al [8] attempted to deplete GSH levels by administering 2 mmol/kg of buthionine sulfoximine (BSO) 2 hours before acetaminophen. BSO [11], an analogue for glutathione synthetase, binds and irreversibly inactivates this enzyme which is the final enzyme in GSH synthesis. The rapid turnover of cellular GSH, ensures that enzyme inactivation leads to a rapid reduction in the hepatocyte GSH concentrations. Kelly et al [8] showed that BSO administration to mice decreased hepatic GSH stores to 15% to 20% of normal within 2 hours. They showed greater liver damage in the BSO group in terms of alanine aminotransferase activity, prothrombin time, bilirubin level, and histological characteristics. Miller et al [12] administered phenobarbital to induce cytochrome P-450 enzymes and thus potentiate subsequent acetaminophen toxicity. This increased liver damage and decreased animal survival.

There has been limited success in the development of suitable large animal models of acetaminophen induced liver injury. In the study described here we used bed-side biochemical and invasive haemodynamic monitoring during acetaminophen-induced liver damage in a reproducible and controlled manner. Using this approach we markedly reduced previously reported problems, i.e., methaemoglobinaemia and cardiovascular collapse, and produced a large animal model which reliably reproduces the syndrome of ALF observed in clinical practice.

## Methods

Animal experiments were performed in accordance with the Home Office regulations under the Animal (Scientific Procedures) Act 1986 as per Project licence 60/2389.

All animals received humane care and study protocols complied with our institution's guidelines.

13 Large White pigs (median body mass 35 kg) were maintained under. Anaesthesia was induced with Ketamine and Midazolam, and subsequently maintained with isoflurane and nitrous oxide according to tidal volume. Background hydration was maintained at a rate of 2 mls/kg/hr using a combination of 0.9% Normal Saline and 5% Dextrose according to electrolyte results from arterial blood gas sampling and urine output. Boluses of colloid (Gelofusine) were administered for episodes of hypotension. Heater pads were used to ensure that core (rectal) temperature was maintained between 37-39°C (the normal temperature range for pigs) [13]. Haemodynamic variables were monitored continuously using a Datex AS/3 monitoring system (Datex Ohmeda, Stirling, UK) and recorded at one minute intervals using the Datex Collect programme on a laptop computer. Measurements were made from arterial, central venous and pulmonary arterial cannulae. The latter allowed hourly left atrial pressure (from pulmonary capillary wedge pressure) and cardiac output (thermodilution) measurements: 10 mls isotonic saline aliquots at 4°C were injected through the injectate port of the pulmonary artery catheter using a closed injectate system (CO-Set plus, Baxter Edwards Critical Care, Irvine, CA, USA). Three measurements were made at random points during the respiratory cycle and an average value taken for each measurement. Intracranial pressure (ICP) was monitored with a subdural pressure transducer (Camino 110-4B, Integra Lifesciences Corp, New Jersey, USA). Correct positioning was confirmed by obtaining an appropriate waveform that fluctuated with ventilation. Urine output was monitored via a urethrostomy. Microbiological testing was performed routinely for all animals.

### Acetaminophen administration

All animals were pre-treated with phenobarbital 20 ml orally per day for 5 days prior to each experiment to induce cytochrome p450 enzymes (n = 13 pigs). Total cytochrome p450 levels were measured using CO difference spectroscopy of sodium hydrosulphite samples [14]. In nine animals intravenous acetaminophen was administered while four animals were used as controls and monitored invasively but did not receive any acetaminophen infusions. An initial loading dose of acetaminophen was administered by intravenous infusion (0.1875 g/kg) followed by an infusion for 12 hours (1.8 mg/kg/min) with the intention of keeping blood acetaminophen concentration between 200-300 mg/l (n = 9 pigs). Acetaminophen levels were monitored by bed-side testing using the Acetasite system (Indiana, USA). Arterial blood gas and lactate levels were measured using a Hewlett Packard I-Stat point-of-care blood analysis system. Methaemoglobin was concurrently measured spectrophotometrically as previously described [15].

Experiments lasted up to 28 hours, and any animals surviving at this time-point were euthanased. Necropsy samples of liver, kidney and brain were then taken. Samples were fixed in formalin and embedded in paraffin. Sections were cut at 5 microns and haematoxylin & eosin stained for routine histological analysis.

### Laboratory measurements

Standard laboratory assays were used for the measurement of liver and renal function, full blood count and coagulation parameters (prothrombin time, factors V, VII, VIII). Serum aspartate aminotransferase, albumin, potassium and creatinine analysis were measured on an Olympus AU2700 automated analyser (Olympus UK Ltd, Watford, UK.) using proprietary diagnostic kits.

### NMR spectroscopy

NMR spectroscopy was used to measure a number of amino acids in serum to calculate Fischer's ratio [16], which is the ratio of the aromatic amino acids (tyrosine and phenylalanine) to branch chain amino acids (leucine, isoleucine and valine). Reversal of Fischer's ratio is a reliable marker of development of encephalopathy in an anaesthetised model. Samples for NMR spectroscopy were prepared and analysed as described previously [17].

### Statistical analysis

Results are presented as means ± S.E.M, as analysis demonstrated the data to be normally distributed. Significance of the differences between means was assessed using the two-sided t test. Two-sided t test was performed to determine the statistical difference after 24 hours between the two groups. Values of p < 0.05 were considered significant.

## Results

Cytochrome p450 levels measured in homogenised liver tissue from phenobarbital pre-treated animals (n = 3 pigs)were significantly higher than in animals which had not received phenobarbital (300 +/- 86 vs 100 +/- 31, p = 0.01) pmol/mg protein. All animals in this study subsequently received phenobarbital

### Acetaminophen toxicity and methaemoglobinaemia

**Figure 1:**
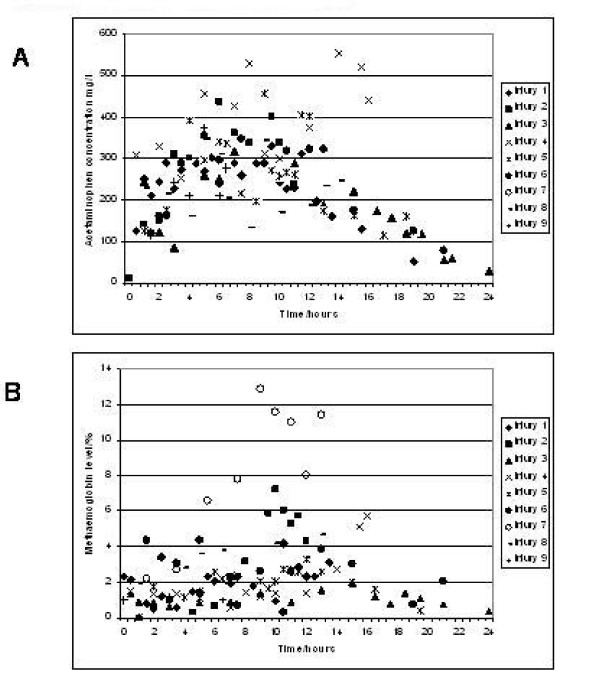
**Acetaminophen concentrations (A) and Methaemoglobin levels (B) in pigs following acetaminophen administration. **Panel A. Acetaminophen levels for individual pigs are noted, with the corresponding methaemoglobin levels illustrated in panel B. Acetaminophen levels of between 200 300 mg/l were aimed for although even with frequent measurements and reductions in the infusion rate there were rises often to 400 mg/l. Prolonged elevation of levels above 300 mg/l did lead to a rise in methaemoglobin. For pig 7 facilities to measure acetaminophen levels were unavailable and this led to a marked rise in methaemoglobin levels.

Acetaminophen was administered by intravenous infusion for 12 hours with the intention of maintaining blood levels between 200-300 mg/l. By accurate monitoring of acetaminophen and methaemoglobin levels this was largely accomplished (figure 1a) although in two (of nine) animals levels exceeded 400 mg/l. The consequence of high acetaminophen levels, namely an increasing methaemoglobin percentage, is demonstrated in figure 1b. In injury 7 we were unable to measure acetaminophen levels, and despite following a previously calculated infusion pattern the pig developed severe methaemoglobinaemia (peak of 13%). This reinforces the importance of close monitoring of acetaminophen levels. In general, methaemoglobinaemia (>4%) occurred approximately 2-3 hours after acetaminophen levels exceeded 350 mg/l. The finding of other groups, i.e., that brown blood discolouration presages the development of methaemoglobinaemia was not confirmed in the current study. Similarly the use of methylene blue (MB) which has previously been described as an effective treatment for methaemoglobinaemia, was associated with death within 5 minutes of injection in the single animal of the current study in which it was administered.

### Animal survival

**Figure 2:**
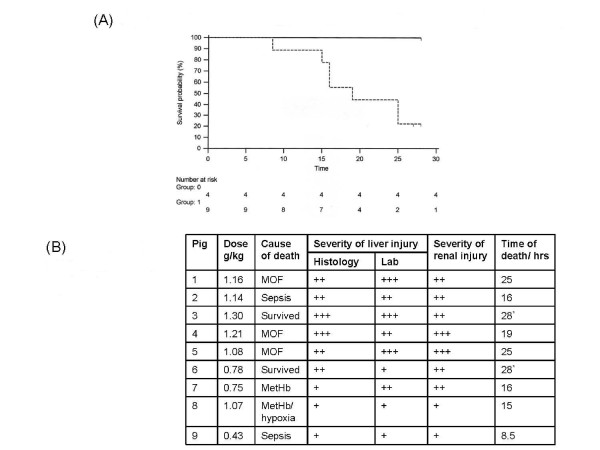
**Kaplan Meier survival curve for control and acetaminophen injured pigs. **(A) The control group (Group 0) as demonstrated by the uninterrupted line all survived, and indeed appeared healthy when euthanased. The injury group (Group 1) as demonstrated by the interrupted line died as denoted in the Kaplan-Meier curve. Two animals* were euthanased at 25 hours as they appeared particularly unwell, and another further two were euthanased at the end of the experiment with no obvious ill effects from acetaminophen administration. (B) This table outlines the likeliest cause of death along with a correlation with the amount of liver and renal injury. In most cases the cause of death was multi-organ failure (MOF) with evidence of hypotension, marked oedema, oliguria and ventilatory difficulties. In two animals organisms were identified in routine blood cultures. In pig 2 a cavitating lung abscess was identified although this was in addition to liver and renal injury. However in pig 9 Pseudomonas paucimobilis was identified in blood cultures with little evidence of significant renal or liver damage. +++ covered severe hepatic coagulative necrosis, ++ covered moderate hepatic coagulative necrosis and + covered mild hepatic coagulative necrosis. Similarly the scoring scale for renal injury covered the range from mild (+) to moderate (++) to severe (+++) tubular necrosis. Pig 7 developed significant methaemoglobinaemia (>10%). Administration of Methylene blue led to circulatory collapse and death within 5 minutes. Pig 8 had moderately elevated levels of methaemoglobinaemia (>4%), and shortly before death dropped its oxygen saturations to 40%. No additional contributory cause to death was identified.

Control pigs (n = 4) survived the 28 hour anaesthetic without complications, whilst only two of the nine injured pigs survived to this time-point (see Figure 2a). Two of the animals which received acetaminophen were critically unwell, and were euthanased at 25 hours. Three pigs developed a syndrome of multi-organ failure (MOF) before death, which is in keeping with the clinical condition [4]. Failure of two or more organ systems was required for MOF to be confirmed: most commonly these were arterial hypotension (refractory to fluid resuscitation) and ventilatory failure. Sepsis was confirmed in two pigs by blood culture (Klebsiella pneumoniae and Pseudomonas paucimobilis). Refractory hypotension and/or difficulties in ensuring successful ventilation led to a joint decision to euthanase pigs.

### Cardio respiratory evaluation

**Figure 3:**
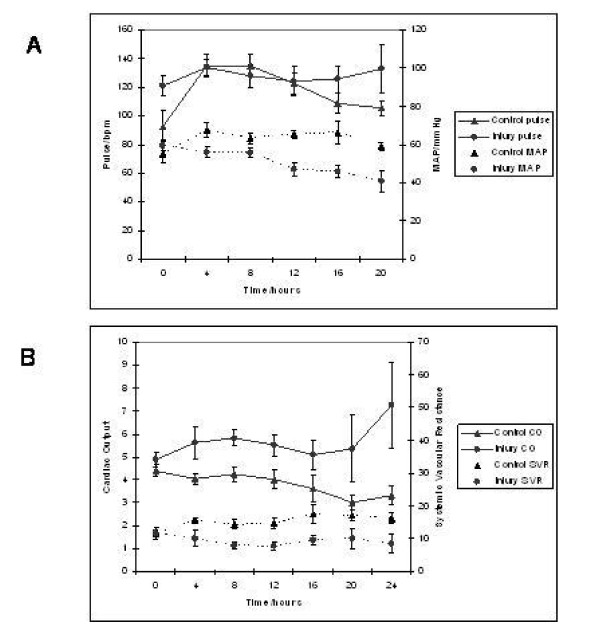
**Profile of Pulse, Mean Arterial pressure, Cardiac Output and Systemic Vascular Resistance in control and acetaminophen injured pigs. **Chart A demonstrates a progressive increase in pulse and concomitant reduction in MAP following liver injury. Furthermore, chart B demonstrates a marked increase in cardiac output (CO) and decreased systemic vascular resistance (SVR) following liver injury. These haemodynamic changes are in keeping with the multi-organ failure seen in acute liver injury.

**Figure 4:**
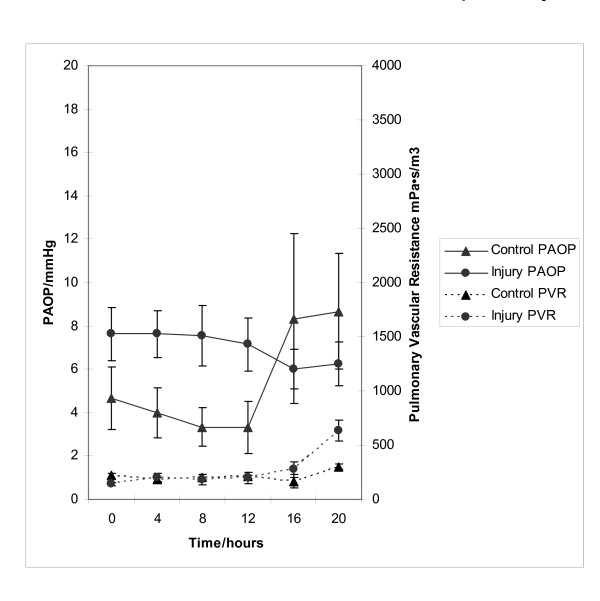
**Profile of Pulmonary Artery Occlusion Pressure and Pulmonary Vascular Resistance in control and acetaminophen injured pigs. **Fluid resuscitation was guided by Pulmonary Artery Occlusion Pressure (PAOP), and therefore this was similar between the two groups. Pulmonary Vascular Resistance (PVR) became noticeably higher in the acetaminophen injured pigs. This was also seen in an increased ventilatory requirement in the latter stages of the injury experiments.

Figures 3a and 3b demonstrate that injured pigs (as opposed to uninjured pigs) developed the typical haemodynamic pattern of acute liver injury characterised by arterial hypotension (40.8 ± 5.9 vs 59 ± 2.0 mmHg, p = 0.16) (MAP, mean arterial pressure), increased cardiac output (CO) (7.26 ± 1.86 vs 3.30 ± 0.40 l/min, p = 0.01) and decreased systemic vascular resistance (SVR) (8.48 ± 2.75 vs 16.2 ± 1.76 mPa·s/m^3^, p = 0.03) that was unresponsive to fluid infused in an attempt to maintain normal pulmonary arterial occlusion pressures (PAOP) (figure 4). Positive pressure lung ventilation was associated with a reduction in total compliance as injury progressed and the pulmonary vascular resistance (PVR) (636 ± 95 vs 301 ± 26.9 mPa·s/m^3^, p < 0.01) increased in this group (figure 4). This may have indicated the onset of an adult respiratory distress syndrome (ARDS)-like picture.

### Laboratory analysis

**Figure 5:**
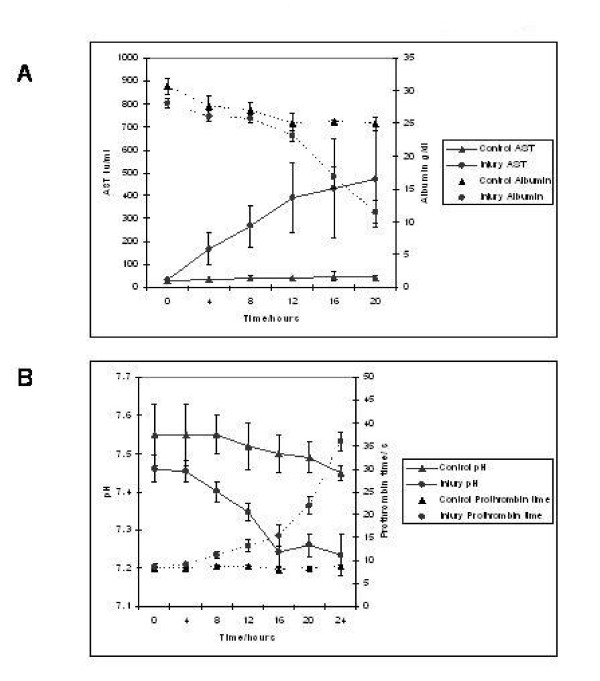
**Profile of serum AST, serum Albumin, arterial pH and plasma Prothrombin time in control and acetaminophen injured pigs. **Panel A depicts the increase in serum AST and concomitant reduction in serum albumin in acetaminophen injured pigs. This pattern of liver injury was also seen in panel B, where arterial pH drops and Prothrombin time increases.

**Figure 6:**
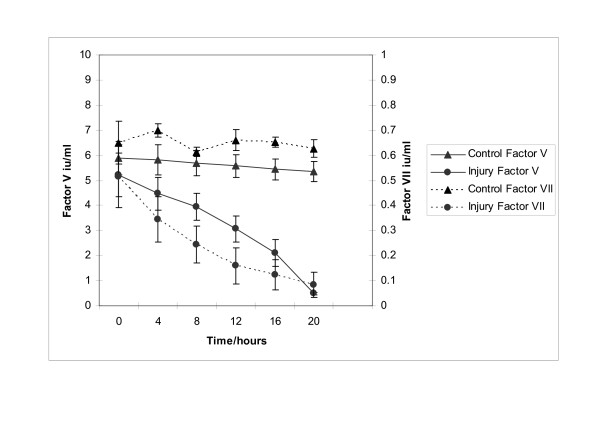
**Profile of the plasma coagulation factors V and VII in control and acetaminophen injured pigs. **The levels of plasma coagulation factors V and VII can be seen to markedly reduce in acetaminophen injured pigs in keeping with the previously observed prolongation of prothrombin time.

Biochemical analysis revealed a marked increase in serum AST (471.5 ± 210 vs 42 ± 8.14, p = 0.03) in conjunction with a marked reduction in serum albumin (11.5 ± 1.71 vs 25 ± 1 g/dL, p = 0.03) in injured animals (figure 5a). AST rather than ALT was used to quantify liver damage as the assay employed was unable to measure porcine ALT from homogenised porcine liver tissue. This hepatic dysfunction was confirmed by pH (7.23 ± 0.05 vs 7.45 ± 0.02, p = 0.02) and prothrombin time (36 ± 2 vs 8.87 ± 0.33 seconds, p = 0.002) (figure 5b). Arterial pH decreased significantly in the injured group in parallel with increasing prothrombin times (to a mean of 36 seconds at 24 hours). The liver components of prothrombin time, factors V and VII were (figure 6a) to decrease after injury to levels of 9.3% and 15.5% of normal values respectively. Factor VIII levels remained unchanged indicating that Disseminated intravascular coagulation (DIC) was not the main cause for the altered levels of other coagulation factors.

**Figure 7:**
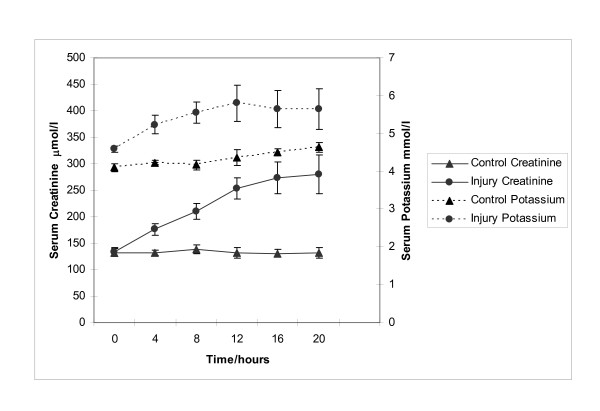
**Profile of serum Creatinine and Potassium in control and acetaminophen injured pigs. **This panel depicts the development of renal injury in acetaminophen injured pigs as assessed by serum Creatinine and Potassium.

In addition to liver injury there was evidence of renal impairment as shown in figure 7 where serum creatinine rises above 250 μmol/l in injured animals, although serum potassium remains below 6 mmol/l. Mean levels of creatinine and potassium were 280.2 ± 36.5 vs 131.6 ± 9.33 μmol/l (p = 0.002) and 5.65 ± 0.53 vs 4.6 ± 0.12 mmol/L (p = 0.27) respectively.

### Evidence of encephalopathy: Fischer's ratio

**Figure 8:**
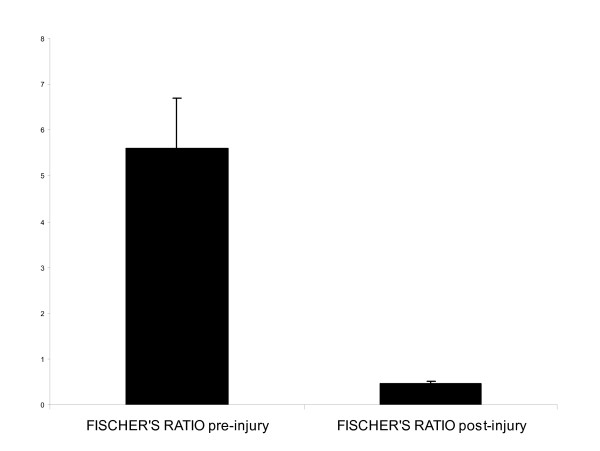
**Fischer's ratio in acetaminophen injured pigs. **Fischer's ratio, a ratio of the concentrations of aromatic amino acids (tyrosine and phenylalanine) to branch chain amino acids (leucine, isoleucine and valine) is demonstrated in the panel. There was no change in the control animal group, but after liver injury there was a marked reduction.

In the liver injury group there was a significant increase in tyrosine (0.03 ± 0.004 vs 0.40 ± 0.02) (p < 0.008) and phenylalanine concentrations (0.05 ± 0.004 vs 0.71 ± 0.08) (p < 0.001) by the end of the experiment. There were no statistically significant changes in leucine levels (0.17 ± 0.02 vs 0.175 ± 0.02) although isoleucine increased significantly (0.08 ± 0.01 vs 0.2 ± 0.01) (p < 0.05) while valine levels decreased (0.2 ± 0.02 vs 0.13 ± 0.01) (p < 0.01). Fischer's ratio (BCAA/AAA) was 5.6 ± 1.1 at the beginning and 0.45 ± 0.06 at the end of the study (see Figure 8). The ratio was unchanged in uninjured pigs.

### Intracranial pressure (ICP) measurements

Intracranial pressure did not alter in any of the control or injured animals at any time during the experiment. Mean levels were 8 mmHg. Cerebral perfusion pressure was less in injured animals, principally because of arterial hypotension; this was not associated with obvious cerebral histopathology.

### Histology

**Figure 9:**
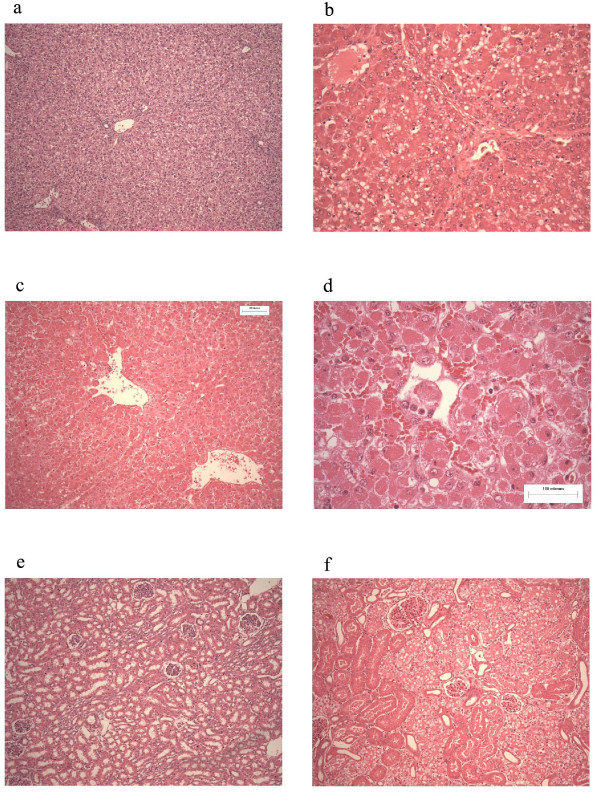
**Histology. **This figure depicts representative photographs of liver and renal sections. Panel a, represents liver tissue taken from a control pig. Panel b represents liver tissue taken from pig 4 and demonstrates moderate injury. There is diffuse microvesicular change, with moderately severe centrilobular necrosis. Panels c and d (higher power) represents liver tissue taken from pig 7 demonstrating more severe coagulative centrilobular necrosis. Panel e represents renal tissue taken from a control pig. Panel f represents liver tissue taken from pig 7 demonstrating severe vacuolar injury to the cortical tubules in keeping with the development of acute tubular necrosis.

Histological analysis of liver tissue revealed perivenular and mid-zone confluent hepatocytic necrosis in the worst-affected animals, with microvesicular change in preserved viable parenchyma. Other animals developed less injury as judged by the extent of necrosis, but qualitatively similar patterns (see Figure 9) were seen. Four animals demonstrated moderately severe liver injury, 2 developed severe coagulative necrosis and 3 developed mild (no zonal confluent necrosis) liver injury. Similarly, renal histopathology of seven pigs revealed severe acute tubular injury as judged by the prevalence of vacuolar change to the cortical tubular epithelium.

## Discussion and Conclusions

The major role for an ALF model is to provide a more controlled experimental environment than currently exists [18]. An ideal model should satisfy the following criteria: 1) induced hepatic failure should be reversible; 2) the features of liver damage should be reproducible; 3) selective damage should lead to fatal liver failure over a period similar to that encountered in the human condition; but 4) with sufficient time to allow the option for successful treatment; 5) the model should be large enough to accommodate anthropocentric therapies; and 6) the toxins used should not be hazardous to laboratory personnel. A pig model would be preferable as it has the potential to fulfil all of these criteria, and also importantly shares very similar liver metabolic function with human liver [19].

The porcine model described here develops most of the signs typifying acute liver injury/failure, namely: tachycardia, hypotension, increased cardiac output, decreased systemic vascular resistance, coagulopathy, renal failure and altered hepatic biochemistry. Consequently, it is the only animal model of acetaminophen-induced liver injury large enough to allow monitoring at a level equivalent to intensive care. Whilst the outcome of acetaminophen-injured pigs is relatively reproducible there still remain some inherent variations highlighting the difficulty of such studies. This reinforces the need to perform large studies when using such animals to ensure that individual variations can be accounted for.

Our model showed the typical increase in cardiac output and reciprocal reductions in SVR found in the clinical setting. Of note this is accelerated within the porcine model, starting within 16 hours of the onset of liver damage. This is likely a reflection of pre-treatment with phenobarbitone. The increased PVR in the context of normal PaO_2 _values was in keeping with the increased requirement for positive end-expiration pressure (PEEP) during ventilation. This may have indicated the onset of an ARDS-like picture, although lung histology would be important to support this conclusion. That PAOP did not increase suggests that the ventilatory changes were not associated with pulmonary oedema secondary to left ventricular failure. A non-significant reduction in PVR Index after liver injury has been described [20]. The reason for this difference is unclear. Acute lung injury is common in patients with acetaminophen-induced fulminant hepatic failure and is associated with systemic circulatory failure, cerebral oedema and high mortality.

The marked reduction in Factor V and VII levels in the current model were consistent with severe liver injury. The Clichy criteria [21] deemed a reduction of Factor V levels to <20-30% as the trigger for liver transplantation; this was the case in most animals in this experiment. The absence of changes in Factor VIII levels indicate DIC was not the cause of altered levels of other coagulation factors.

An increase in AST levels indicated the development of liver necrosis, although these were not as high as are seen in patients. Similarly, a rise in bilirubin did not occur. This may reflect a species-specificity of the assay used, or insufficient time for bilirubin to rise. It is possible that there are subtle variations in this model compared to humans in that some systemic features prevail over a liver focused pattern of injury. Liver tissue analysis revealed severe coagulative necrosis in some animals with moderate and mild injury in others. This supports the probability that significant liver injury accounts for the clinical syndrome reported here. Although there is some variability in the liver injury seen in this model, there is still significant liver injury in the majority of animals. Indeed, this variation closely mimics what is seen clinically.

There are several explanations for the failure to observe changes in ICP. The time course may have been too brief. However, a raised ICP is not always encountered in the clinical setting, or only in association with other factors such as sepsis. It may also reflect differences in the pathophysiology of drug-induced ALF as opposed to ischaemic models of ALF. Measurements of arterial ammonia, cerebral blood flow or the use of cerebral microdialysis may help to clarify intracranial effects in future studies. Biochemical analysis suggested that animals may have been developing encephalopathy, although in the absence of ICP changes and with general anaesthesia it is difficult to be certain. Fischer's ratio [16], the ratio of the aromatic amino acid (AAA; tyrosine and phenylalanine) to branch chain amino acid (BCAA; leucine, isoleucine and valine) concentrations has a value of 3 to 4 in clinical liver failure but when this ratio is reversed and is in absolute figures <1.4 then most patients develop hepatic encephalopathy.

There was evidence of marked renal impairment, although fatal hyperkalaemia was not observed. Renal dysfunction is an important feature of acetaminophen induced liver failure [22], and the renal histology in treated animals was consistent with a toxic injury rather than the distinctive pattern of frank outer medullary tubular necrosis encountered with ischaemia, i.e., critical hypotension.

### Methaemoglobinaemia

This study demonstrates the importance of continuous monitoring of acetaminophen levels, the one animal from which it was withheld died from methaemoglobinaemia. The amount of acetaminophen required to maintain levels between 200-300 mg/l for 12 hours was calculated beforehand, but it was necessary in every case to reduce the infusion rate (and the total dose administered) as acetaminophen levels exceeded this range. Levels greater than 250 mg/l increased serum methaemoglobin levels, although this usually resolved by stopping the acetaminophen infusion which allowed plasma levels to fall. Acetaminophen and its intermediates oxidise haemoglobin to methaemoglobin, which is unable to carry oxygen. Although it occurs in human beings, it is less marked than in cats, dogs, and pigs. In some species, methemoglobin is reduced to hemoglobin by the methemoglobin reductase using reduced glutathione (GHS) as a substrate. GSH itself is recycled by the glutathione reductase, using reduced nicotinamide-adenine dinucleotide phosphate (NADPH) from the pentose phosphate pathway. Due to the glucose impermeability of porcine red blood cells (compared with rats and rabbits) there is a diminution in generation of NADPH and hence decreased reduction of methaemoglobin [23]. Levels of methaemoglobinaemia in pigs [24] administered acetaminophen are only one-half those reported in cats [25] and dogs [26]. There are several measures to control methaemoglobin levels in animal models. Kelly et al [8] suspected high plasma acetaminophen levels were responsible for lethal methaemoglobinaemia and so tried to prevent them. Not having access to bedside testing and thus being unable to prevent high acetaminophen levels they administered MB at 10 mg/kg if the blood appeared brown on visual inspection. The usefulness of this strategy was not confirmed in the current study.

Methylene blue is the recognized clinical treatment for methaemoglobinaemia and acts by reducing methaemoglobin back to haemoglobin. Its action depends on the availability of adequate NADPH concentrations within the erythrocyte; a deficiency of NADPH availability leads to further methaemoglobinaemia. On the single occasion it was used in the current study the animal died of cardiovascular collapse 5 minutes later.

### Presence of anaemia

Miller et al [12] found that the haematocrit fell rapidly (25% decrease from the initial packed cell volume) in sixty percent of animals 1 to 2 hours before death. The cause of anaemia in their study was unclear, and may reflect extravascular haemolysis within the spleen. The haemoglobin nor haematocrit levels did not fall in either the control nor the treatment groups in the current study.

In summary, we have described the first intensively monitored porcine model of acetaminophen-induced severe liver injury which displays most of the features seen in the human condition. This large animal model can play an important role in the evaluation of the effectiveness of liver support systems, as well as providing us with the ability to develop a much better understanding of the pathophysiology of this devastating clinical syndrome.

## Competing interests

The authors declare that they have no competing interests.

## Authors' contributions

PNN, NCH, CF, LJN, AL, TK carried out the studies and drafted the manuscript. REC carried out the studies. CB carried out histological analysis and drafted the manuscript. FH carried out the biochemical analysis and drafted the manuscript. KD carried out the NMR analysis and helped draft the manuscript. LJN, PNN, PCH, JNP conceived of the study, and participated in its design and coordination and helped to draft the manuscript. All authors read and approved the final manuscript.

## Pre-publication history

The pre-publication history for this paper can be accessed here:

http://www.biomedcentral.com/1471-230X/10/34/prepub
